# The Application of Unsupervised Clustering Methods to Alzheimer’s Disease

**DOI:** 10.3389/fncom.2019.00031

**Published:** 2019-05-24

**Authors:** Hany Alashwal, Mohamed El Halaby, Jacob J. Crouse, Areeg Abdalla, Ahmed A. Moustafa

**Affiliations:** ^1^Department of Computer Science and Software Engineering, College of Information Technology, United Arab Emirates University, Al-Ain, United Arab Emirates; ^2^Department of Mathematics, Faculty of Science, Cairo University, Giza, Egypt; ^3^Brain and Mind Centre, The University of Sydney, Sydney, NSW, Australia; ^4^School of Social Sciences and Psychology, Western Sydney University, Sydney, NSW, Australia

**Keywords:** clustering, neurological diseases, Alzheimer’s disease, unsupervised learning, machine learning techniques

## Abstract

Clustering is a powerful machine learning tool for detecting structures in datasets. In the medical field, clustering has been proven to be a powerful tool for discovering patterns and structure in labeled and unlabeled datasets. Unlike supervised methods, clustering is an unsupervised method that works on datasets in which there is no outcome (target) variable nor is anything known about the relationship between the observations, that is, unlabeled data. In this paper, we focus on studying and reviewing clustering methods that have been applied to datasets of neurological diseases, especially Alzheimer’s disease (AD). The aim is to provide insights into which clustering technique is more suitable for partitioning patients of AD based on their similarity. This is important as clustering algorithms can find patterns across patients that are difficult for medical practitioners to find. We further discuss the implications of the use of clustering algorithms in the treatment of AD. We found that clustering analysis can point to several features that underlie the conversion from early-stage AD to advanced AD. Furthermore, future work can apply semi-clustering algorithms on AD datasets, which will enhance clusters by including additional information.

## Introduction

There has been an increasing interest in the medical community to use machine learning techniques for disease diagnosis (Kononenko, 2001). This is due to the increases in availability of medical datasets, such as Twinanda et al. (2017), Srivastav et al. (2018), Alzheimer’s Disease Neuroimaging Initiative (ADNI), and UC Irvine Machine Learning Repository, among others. The accumulation of large datasets has become more feasible recently due to the advancements in hardware (fast, cheap computers), the availability of public and private medical and healthcare datasets, and machine learning classification and clustering methods.

Supervised learning is the process of learning (approximating) a mapping function from a set of input variables to a target variable. The term “supervised” here refers to the training process of the algorithm being supervised by having the correct answers (i.e., we know what the target outcome is). However, when one only has a set of variables and no corresponding output variables (i.e., the data are unlabeled), then the learning process is called unsupervised. Thus, in unsupervised learning, there are no correct answers for the training procedure to learn from and the learning algorithm is left to discover the structures in the datasets. One of the most important unsupervised learning techniques is clustering, which is the process of partitioning a set of data points according to some measure of similarity (e.g., distance). The goal of clustering is to reveal subgroups within heterogeneous data such that each individual cluster has greater homogeneity than the whole (Eick et al., 2004). Table 1 summarizes the different types of machine learning methods and some of their real-world applications. In many applications, obtaining labeled data is often difficult, costly, and/or time-consuming, while collecting unlabeled data may be relatively easy. Such cases result in a dataset consisting of a large number of unlabeled variables and a small set of labeled variables. Semi-supervised learning uses both labeled and unlabeled data to improve the accuracy of the learning model.

**Table 1:** Types of machine learning methods.

**Learning type**	**Supervised**	**Unsupervised**	**Semi-supervised**
Type of data	Data points have labels.	Data points do not have corresponding labels.	A subset of the data points is labeled.
Learning process	Analyzing the training data to learn a function that can be used for predicting the labels of new examples.	Modeling the structure or the distribution of the data in order to find patterns and gain new insights from the data.	Utilizing unlabeled data with labeled data to learn better models.
Applications	Fraud detection, detecting spam emails, predicting real estate prices.	Clustering customers' data and market segmentation, learning rule associations, image segmentation, gene clustering.	When it is expensive to annotate every data point (e.g., using humans), this type of learning is suitable. Examples: web content classification, medical predictions.

*Firstly, the nature of the data is stated, then the objective of the type of learning is discussed, and finally some real-world examples are mentioned*.

Several studies have used clustering methods to facilitate the diagnosis of several disorders (Vogt and Nagel, 1992; Nugent and Meila, 2010; Li and Zhu, 2013; Nithya et al., 2013; Wiwie et al., 2015). For example, clustering techniques have been applied to the diagnosis of breast cancer (Chen, 2014), Parkinson's disease (Polat, 2012; Nilashi et al., 2016), headache (Wu et al., 2015), mental health and psychiatric disorders (Trevithick et al., 2015), heart and diabetes diseases (Yilmaz et al., 2014), and Huntington's disease (Nikas and Low, 2011), among many others.

Alzheimer’s disease (AD) is one of the most common neurodegenerative diseases, particularly in old age (Ryu et al., 2010), and is among the most common causes of dementia in senior individuals (Ryu et al., 2010; Cuingnet et al., 2011). AD leads to structural and functional loss of neurons in the cortex and hippocampal regions, among other brain areas. A number of studies in the past 20 years have pointed out possible biomarkers for the diagnosis of AD, including brain atrophy revealed by magnetic resonance imaging (Mueller et al., 2006; Seppi and Poewe, 2010).

## Method

In this paper, we summarize prior studies that use clustering methods on AD datasets to gain more insights into the disease's nature, diagnosis, and progression. In the following sections, we describe the most common clustering algorithms and their application on AD datasets in the literature. A computer search was carried out, containing the clustering and AD. This search was performed in PubMed and Google Scholar.

## Clustering Algorithms

### k-Means

The k-Means clustering algorithm (Forgy, 1965) is a classical unsupervised learning method. This algorithm takes *n* observations and an integer *k*. The output is a partition of the *n* observations into *k* sets such that each observation belongs to the cluster with the nearest mean. The following steps summarize the operations of k-Means.

Initialize *k* cluster centers. In practice, this can be done by either randomly selecting *k* center

1. points from the *n* observations or random generation of *k* center points.
2. Calculate the distance between each observation and the cluster centers.
3. Assign each point to the cluster whose distance from its center is minimum of all the cluster centers.
4. Recompute the positions of the *k* centers as the cluster mean.
5. Recompute the distance between each data point and the newly computed centers. Repeat steps 3 and 4 until all data points are assigned to the same cluster (data points do not move).

The choice of *k* is usually influenced by prior knowledge regarding the nature of the data or by using clustering validity measures.

Escudero et al. (2011) investigated how applying k-Means clustering to a subject's medical history may shed light on the likelihood of conversion from mild cognitive impairment (MCI) to AD. The dataset used was obtained from the ADNI database and consists of 375 subjects. The selected features included the number of ApoE s4 alleles, ADAS-Cog (Alzheimer’s Disease Assessment Scale-Cog), Mini-Mental State Examination (MMSE) scores, MRI (magnetic resonance imaging), and CSF (cerebrospinal fluid) data from cognitively normal (CN), MCI, and AD individuals. The authors tested the potential of how having the following five sets of features can better diagnose AD: (1) ADAS-Cog, MMSE, and ApoE genotype obtained from a blood sample; (2) CSF; (3) MRI; (4) CSF and MRI; and (5) all of the above features. The first analysis involved clustering the subjects according to each of the five scenarios (i.e., using only a subset of the variables based on the set of features described above) using k-Means and approximating the occurrence of the medical history of AD in each set. More than 69% of the AD subjects and about half of the MCI individuals were always assigned to the pathological bioprofile.

In the second analysis, k-Means was applied to the CN and AD subjects, and the obtained clusters were used to split the MCI subjects into CN-like and AD-like, that is, which MCI subjects may stay as healthy individuals and which may convert to AD. Next, the rate of decline to AD was used to evaluate the utility of this clustering algorithm in the early diagnosis of AD at the MCI stage. The fifth set of features (which included all features) provided larger differences between the evolution of CN-like and AD-like subjects at the 12-month follow-up. The number of subjects assigned to CN-like and AD-like was 82 and 96, respectively. This indicates that the combination of all clinical tests and biomarkers outperformed using any of them in isolation.

In a recent study, Tosto et al. (2016) applied k-Means clustering algorithm on a dataset of 3,502 patients with AD with longitudinal assessments from the National Alzheimer’s Coordinating Center database, with 394 providing neuropathological data. The authors were interested in examining subgroups of patients with variable trajectories of extrapyramidal sign progression (which include movement disorders such as postural instability, tremors and rigidity, body restlessness, and abnormal gait, among others) and their clinical and neuropathological correlates. Tosto et al. (2016) observed the following three clusters of extrapyramidal sign progression: no/low (*n* = 1,583), medium (*n* = 1,259), and high (*n* = 660) extrapyramidal burden. The high extrapyramidal cluster had greater cognitive and neuropsychiatric impairment (particularly hallucinations), relative to the other clusters. Moreover, despite the three clusters having similar AD pathology, the high extrapyramidal burden cluster had a significantly greater number of patients diagnosed with dementia with Lewy bodies.

In another recent study, Price et al. (2015) recruited participants with AD or vascular dementia and collected MRI measures of infarction, whole brain volume, and leukoaraiosis (LA), as well as neurocognitive measures in all participants. A k-Means cluster analysis derived three cluster-groups characterized by single-domain amnestic (*n* = 41), single-domain dysexecutive (*n* = 26), and multi-domain (*n* = 26) phenotypes. The multi-domain patients scored worse on language measures than the other clusters, yet they were equally impaired on tests of memory when compared to the amnestic group. The three cluster-groups were relatively dissociable in neuroradiological parameters, in which the amnestic and multi-domain clusters had smaller hippocampal volume than the third cluster, while the single-domain dysexecutive cluster had greater deep periventricular (i.e., between periventricular and infracortical regions) and whole brain LA. The volume of the caudate and lacunar infarction did not differ between the three clusters. There was a negative association between the volume of the caudate nucleus and total LA in the dysexecutive and multi-domain clusters. These results suggest the existence of neuroradiological heterogeneity between patients diagnosed with AD/vascular dementia spectrum dementia.

### k-Means-Mode

This algorithm can deal with both numeric (continuous) and categorical data. Each cluster center is an array of means and modes for continuous and categorical attributes, respectively. The steps of the algorithm is similar to that of the classical k-Means; the means and modes are calculated for each cluster as previously stated, and then each point is moved to the cluster with minimum distance. For continuous features, Euclidean distance is often used, and for discrete features, Hamming distance is often used.

Paul and Hoque (2010) have applied the k-Means-Mode clustering algorithm to medical datasets to predict the likelihood of diseases. The likelihood of the disease in a cluster is defined as the number of patients that have the disease divided by the total number of points in the clusters. In other words, it is the probability of finding the disease in the cluster. The average likelihood of all clusters is the actual probability of the disease in the data, which can be found by brute-force methods. Accuracy is the ratio between average likelihood and actual likelihood. Experimental results show that when the algorithm was applied on the Zoo dataset from the University of California at Irvine (UCI) Machine Learning Repository and a diabetes dataset, an accuracy of about 95% is achieved. Other algorithms like k-Means and k-Mode achieved lower than 65% accuracy, suggesting that the k-Means-Mode algorithm is better at clustering data than k-Means and k-Mode algorithms.

### Multi-Layer Clustering

The first step of the multi-layer clustering process is to determine the similarity between each pair of examples. This is done by creating an artificial binary classification problem having the original patient records as the positive example, while negative examples are generated by randomly mixing the values of the attributes of the original examples among themselves. Next, a predictive model is built to distinguish between the positive and negative examples to determine the similarities between each pair of examples. The Random rules algorithm (Pfahringer et al., 2004; Almeida et al., 2013) is applied for each pair of records to construct an example similarity table (EST) where the number of rules covering the pair is calculated. An entry *e*_*i*__, j_ in the table holds the similarity value between the *i*th and the *j*th example. The second step is to calculate the clustering-related variability (CRV) measure for all examples. The single-layer clustering algorithm starts by assigning each example to a single cluster. It then keeps merging the most similar clusters in terms of the cluster CRV score. The procedure stops when no further merge operations are possible; that is, further merges do not result in a smaller CRV score. In situations having more than one attribute layer (multi-layer attributes), the artificial binary classification problem is constructed for each attribute layer and the ESTs are built. As for the algorithm, for each pair of clusters, the potential variability reduction for all attribute layers is computed and the smallest value for each pair is selected. Merging occurs if this value is positive, and if the value is positive for more than one pair, the pair with the largest minimal value is chosen and these clusters are merged.

Gamberger et al. (2016a) applied a multi-clustering method to an AD dataset of both male and female patients comprising 243 biological and clinical features. The clusters obtained showed differences between male and female patient groups, including the existence of two male subpopulations with changes to intracerebral and whole brain volumes. The multi-layer clustering technique was used to deal with layers of attributes; that is, a set of attributes is partitioned into several subsets according to a criterion (e.g., laboratory data features and clinical data features). The multi-layer clustering technique was carried out independently on two groups of 317 female and 342 male patients. The first layer consisted of 56 biological measurements and the second consisted of 187 symptoms and clinical descriptors. The authors reported key differences between male and female populations of patients. For example, in the female population, there were two clusters, while in the male population, there were four, two for patients having major issues with dementia (denoted M1 and M2) and two for patients having mild or no dementia (denoted M0A and M0B). There was one large cluster in the female population, denoted F1, with patients having significant problems with dementia, while patients in the other cluster had mild dementia symptoms (denoted F0). Patients in cluster M2 were found to have higher than average intracranial volume (ICV) and whole brain volumes when compared to cognitively normal male patients. Such a cluster was not observed in the female population. The M0A cluster was similar to cluster F0 in the female population in terms of increased ICV values and biological features, while cluster M0B had smaller than average ICV values. This analysis showed that there are significant gender-specific differences in AD patients and suggests that taking gender into account may have important implications for the treatment of AD.

The same multi-layer clustering algorithm used by Gamberger et al. (2016a) was also used on a dataset of 218 female and 344 male individuals with MCI. The algorithm first builds an EST for each attribute layer and then the tables are used by a bottom-up method to merge similar clusters together until no further merging of clusters is possible. The goal of this study is to find homogeneous groups of MCI individuals in terms of baseline and prognostic features and to discover gender differences within the groups. The algorithm produced a cluster of “slow decliners” (i.e., individuals with MCI that slowly develop dementia symptoms) consisting of 184 subjects that included a subset of MCI individuals that had favorable baseline data and prognosis. Another cluster given by the algorithm, termed “rapid decliners” (i.e., individuals with MCI that rapidly develop dementia symptoms; *n* = 240), consisted of a subset of MCI subjects with a more impaired baseline status and a rapidly progressing longitudinal cognitive course. Moreover, 138 subjects did not fit in either of the two clusters. Males and females in the “rapid decliners” cluster had worse baseline cognitive status and smaller brain volumes than those in the “slow decliners” cluster. The rate of progression from MCI to dementia for females and males in the “rapid decliners” cluster was 69 and 61%, respectively. Conversely, the rate of progression from MCI to dementia for females and males in the slow decliners cluster was 9 and 16%, respectively.

Gamberger et al. (2016b) applied the multi-layer clustering method used by Gamberger et al. (2016a) and Gamberger et al. (2017) to an AD dataset obtained from ADNI. The dataset consists of 187 cognitively normal (CN) subjects, 106 patients with significant memory concern (SMC), 311 patients with early MCI (EMCI), 164 patients with late MCI (LMCI), and 148 AD patients (916 subjects in total). There are two layers that make up the features: layer 1 consists of 10 biological features and layer 2 consists of 23 clinical features. The goal of this study was to find clusters that are as large and homogeneous as possible regarding both biological and clinical features. Three clusters were identified having patients with different levels of dementia. The first cluster, A, contained patients with low volumes of hippocampus, entorhinal cortex, fusiform gyrus, and middle temporal gyrus, as well as small intracerebral and whole brain volumes. The number of subjects in that cluster diagnosed with AD, LMCI, and EMCI were 30, 4, and 1, respectively. Compared to CN subjects, patients in cluster A had 20% lower mean values for fusiform and midtemporal gyrus. Moreover, patients in cluster A had, on average, a 30% smaller entorhinal volume than the CN group. The authors regarded it odd that patients with LMCI and EMCI were assigned to this cluster, yet offered no explanation for this discrepancy. It is quite possible that these individuals may be at risk for converting to AD; this hypothesis should be tested in future work. Further, patients in cluster A showed high Clinical Dementia Rating Sum of Boxes (CDRSB), Alzheimer’s Disease Assessment Scale (ADAS13), and Functional Assessment Questionnaire (FAQ) scores and low Mini-Mental State Examination (MMSE) and Montreal Cognitive Assessment (MoCA) scores, which is consistent with patients suffering from acute dementia. Importantly, the number of AD, LMCI, and EMCI patients in the second cluster, B, was 10, 9, and 2, respectively. Patients in this cluster have, to some extent, had smaller volumes of entorhinal, hippocampus, fusiform, and midtemporal gyrus that are about 20, 20, 10, and 10% (respectively) lower than mean values for CN subjects. However, the intracranial volume and whole brain volume were normal. Subjects in this cluster had a moderate or mild type of AD, which is indicated by a score above 3 in the CDRSB. An interesting feature of patients in cluster B was that the values for cognitive functions self-reported by the patients were higher than those of the other clusters and of the mean values of the entire AD population.

The third cluster, C, included patients with the lowest degenerative changes in the hippocampus, entorhinal, fusiform, and midtemporal gyrus. Moreover, patients in this cluster had high scores of ventricular and whole brain volumes. Cluster C patients had larger mean ventricle volume than CN subjects. The values for the scales of the MoCA, FAQ, fluorodeoxyglucose imaging (FDG), MMSE, and ADAS13 were all intermediate between those of clusters A and B. Cluster C patients also showed impairment, performing the Rey's Auditory Verbal Learning Test (RAVLT), and divided attention.

This study shows that the nature of the cluster of patients having problems with dementia is non-homogeneous. Moreover, cognitively normal subjects are even more non-homogeneous as a population, as the clustering algorithm reported here shows that there are many clusters of controls as well. The number of AD patients assigned to clusters A, B, and C is < 50% of the entire AD population. Another important finding of the current study is the correlation between cognitive impairment and brain atrophy. The presence of degenerative changes of the brain was found in the three derived clusters. The greatest degeneration was found in cluster A and the second greatest degeneration was found in cluster B. The results obtained from cluster C indicate that brain changes are responsible for a significant number of problems with dementia; however, they are not sufficient for AD development.

## Hierarchal Agglomerative Clustering

Hierarchal agglomerative clustering is a bottom-up approach such that each data point begins in a separate cluster, and pairs of clusters at the bottom are merged together as we go up the hierarchy. This method can be summarized as follows:

1. Assign each object to a separate cluster.
2. For each pair of clusters, calculate the pairwise distance. Then, build a matrix whose elements are the distance values computed.
3. Find the pair of clusters with the shortest distance.
4. Merge the identified pair after removing both clusters from the distance matrix.
5. Calculate all distances from this new cluster to all other clusters and update the distance matrix.
6. Repeat these steps until the matrix is reduced to a single element.

There are several distance metrics that can be used (e.g., Euclidean and Manhattan distances); however, the choice of a metric determines the shape of the clusters produced. This is because two clusters can be close to each other according to one metric, but far from each other according to another metric. It is recommended that an exploratory study be conducted on several distance measures and the one that yields the best results according to chosen performance measures is selected. Unlike k-Means, the number of clusters is not determined by the user, and generally, smaller clusters are generated, which can be helpful in many domains.

Noh et al. (2014) collected high-resolution T1-weighted volumetric MRIs from 152 patients in the early stages of AD. A hierarchical agglomerative clustering analysis was applied to measures of cortical thickness in these patients. Three emergent clusters were compared with an age- and sex-matched control group. The first cluster (A) was characterized by bilateral medial temporal-dominant atrophy predominantly involving anterior and posterior cingulate cortices (*n* = 52, 32.4%); the second cluster (B) was characterized by parietal-dominant atrophy involving bilateral parietal areas, precuneus, and bilateral dorsolateral frontal areas (*n* = 28, 18.4%); and the third cluster (C) was characterized by diffuse atrophy, in which almost all association cortices demonstrated atrophy (except for orbitofrontal and occipital areas) (*n* = 72, 47.4%). Patients in the parietal-dominant cluster (B) were younger, had a younger age at onset, and had the highest years of education. Patients in the diffuse atrophy cluster (C) had the lowest mean cortical thickness. Patients in the parietal-dominant cluster scored the poorest across all neurocognitive tests (attention, visuospatial function, memory, and frontal executive tasks) except for language function measures. These results suggest that there is considerable anatomical heterogeneity evident even in early stages of AD, which may indicate multiple disease processes.

Hwang et al. (2016) conducted several analyses on a dataset that includes 77 patients with AD recruited via the ADNI. Patients underwent 3-T MRI, [^18^F]-fluorodeoxyglucose PET, [^18^F]-florbetapir PET, and cerebrospinal (CSF) tests. Hierarchical agglomerative cluster analysis was applied to measures of cortical thickness, and the remaining measures were compared across groups. Consistent with the study by Noh et al. (2014) and Hwang et al. (2016) observed three clusters, dominated by medial–temporal atrophy (19.5%), parietal atrophy (24.7%), and diffuse atrophy (55.8%). The parietal-dominant cluster was younger and showed greater glucose hypometabolism in parietal and occipital cortices, as well as pronounced amyloid-beta accumulation in most brain regions. The medial–temporal dominant cluster had greater glucose metabolism in the left hippocampus and bilateral frontal cortices and poorer performance on memory tests. There were no significant differences in CSF tests between cluster-groups.

Racine et al. (2015) studied a sample of 103 asymptomatic adults with genetic risk and parental family history of AD. Participants underwent [C-11] Pittsburgh Compound B (PiB) amyloid imaging, MRI, lumbar puncture, and neurocognitive assessment at baseline, with 79 participants also undergoing follow-up PiB imaging 2 years later. The hierarchical agglomerative cluster analysis derived four cluster-groups based on three biomarkers, including CSF total-tau, CSF Aβ_42_, and average PiB burden across 8 AD-sensitive regions of interest. All clusters were compared on amyloid accumulation (controlling for PiB baseline, age, sex, and APOE4 status) as well as on cognitive changes on tests of memory and executive control (controlling for baseline scores, age, sex, APOE4 status, education, and duration between testing visits). Cluster 4 showed the greatest AD-like characteristics (low CSF Aβ_42_ and high PiB), with greater amyloid accumulation over 2 years relative to the other three clusters in regions affected by AD (precuneus, posterior cingulate, and lateral temporal and parietal cortices). Moreover, individuals in cluster 4 scored worse than those in cluster 1 on immediate recall and worse than all three clusters on delayed recall. Individuals in cluster 2 scored better than individuals in cluster 3 on delayed recall and better than both clusters 1 and 2 on total recall. These results suggest that clustering at-risk individuals across validated biomarkers may provide novel insights into those at greatest risk for amyloid accumulation and cognitive decline.

Cappa et al. (2014) recruited 23 patients with posterior cortical atrophy (PCA) and 16 patients with dementia of Alzheimer’s type (AD). First, a principal component analysis was used to reduce 15 neurocognitive variables to the following five factors: memory, language, perceptual processes, visuospatial processes, and calculation (addition, subtraction, and multiplication). These factors were then entered into a hierarchical agglomerative cluster analysis. Four clusters were derived and were characterized by visuospatial/perceptual, memory, perceptual/calculation, and language performance. Four clusters were derived, Cluster 1 (*n* = 9, 100% PCA), Cluster 2 (*n* = 10, 20% PCA), Cluster 3 (*n* = 6, 50% PCA), and Cluster 4 (*n* = 14, 64% PCA). The authors noted that AD pathology appears to produce multiple distinct syndromal subtypes involving impairment in memory (classically associated with AD) and visuospatial deficits (classically associated with PCA), as well as in visual perception and language, which may indicate heterogeneity in vulnerability of specific functional networks.

Armstrong and Wood (1994) applied hierarchical cluster analysis to a group of 78 patients with AD. The dataset consisted of 47 neuropathological measures, including the density and distribution of senile plaques and neurofibrillary tangles. The analyses indicated that an initial splitting of the sample could be made, characterizing one large group (68%) who had a relatively small distribution of senile plaques and neurofibrillary tangles across the brain and a second smaller cluster (15%) who had more diffusely spread lesions throughout the neocortex. These clusters could be further divided based on the extent of capillary amyloid angiopathy. Moreover, patients with a limited development of senile plaques, neurofibrillary tangles, and capillary amyloid angiopathy could be further split into an early- and a late-onset group. Patients with familial AD were not assigned to a single cluster; rather, they were distributed across four of the five groups. Some patients with familial AD had unique combinations of pathological features that did not closely resemble the other clusters.

McCurry et al. (1999) recruited a population-based sample of 205 patients with AD from the Alzheimer’s Disease Patient Registry to investigate patterns of sleep problems. The authors applied hierarchical cluster analysis (Lance and Williams, 1967) to patients who were reported to have awakened their caregivers from sleep. They identified one cluster with daytime inactivity but few behavioral problems, another cluster with higher levels of fearfulness, fidgeting and occasional sadness, and a third cluster with multiple behavioral problems that included frequency bouts of sadness, fearfulness, inactivity, fidgeting, and hallucinations. The results demonstrate the heterogeneity of sleep disturbances in AD, which may have implications for the direction of interventions to homogeneous subgroups experiencing similar patterns of sleep problems.

## Discussion

**Figure 1:**
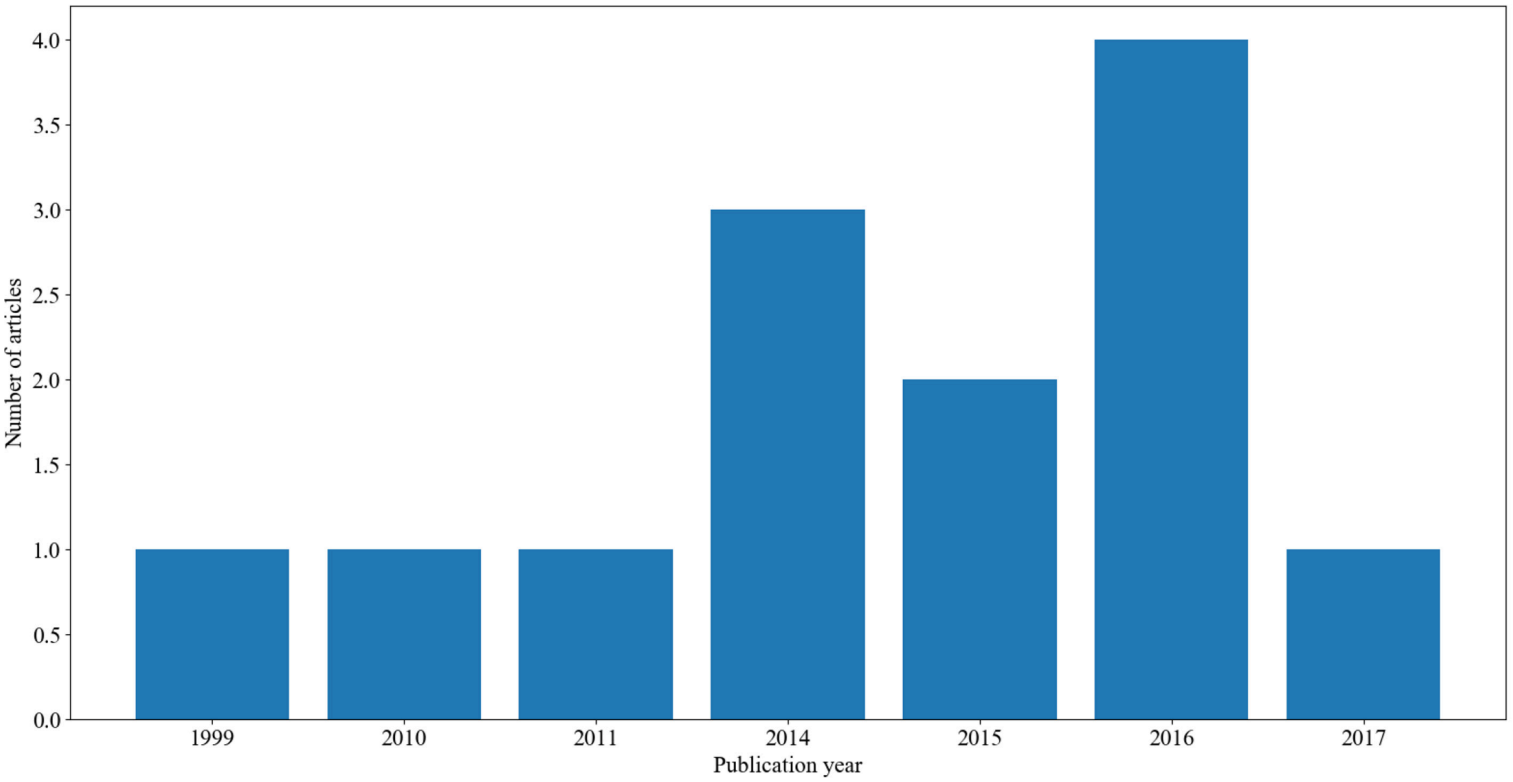
A summary of the number of articles and their corresponding year of publication.

In this study, we were able to identify and review 13 articles that applied clustering methods on mainly AD datasets. To our knowledge, these are the only existing studies on clustering AD datasets. The distribution of these articles over time is presented in Figure 1.

**Figure 2:**
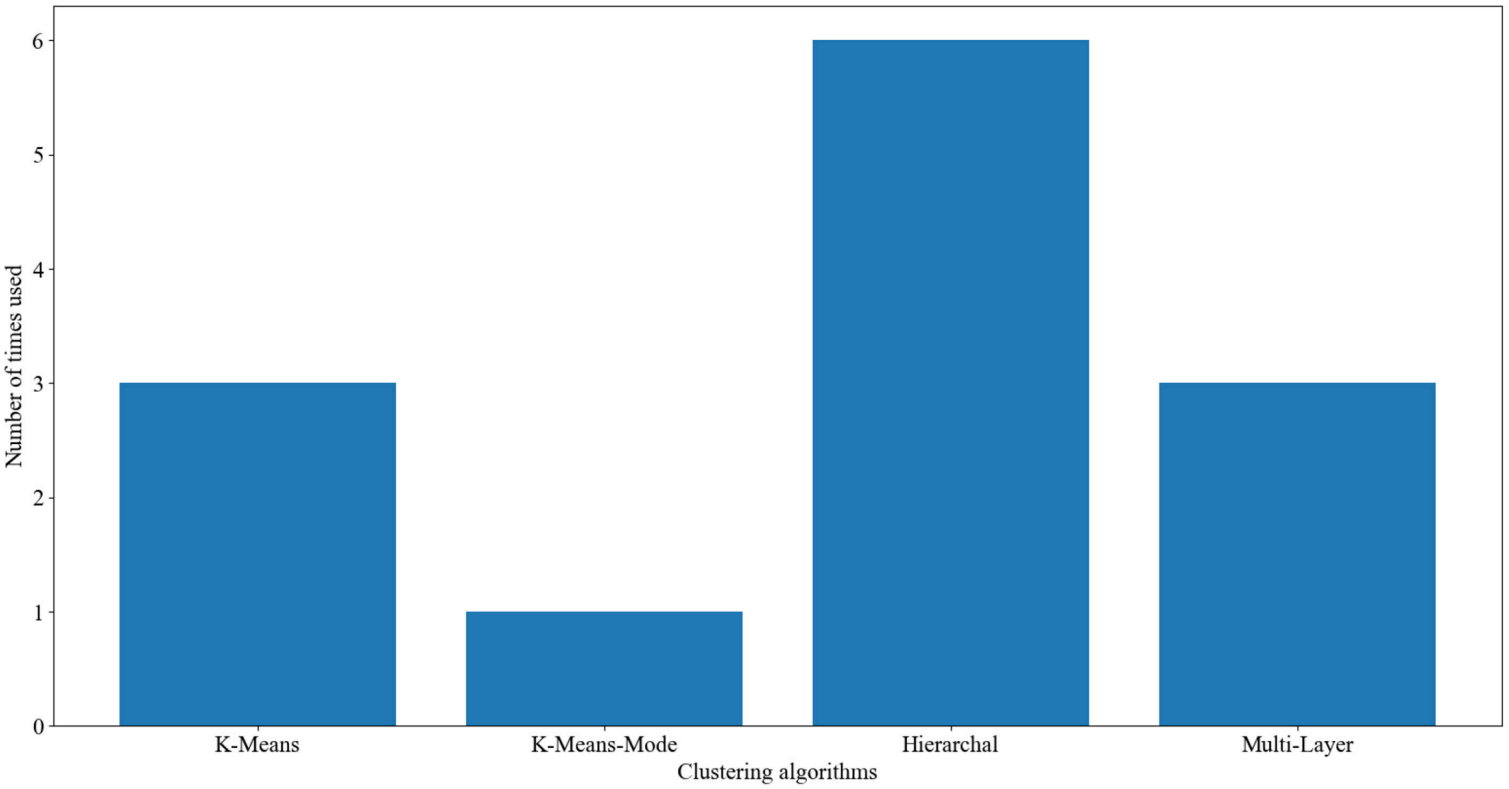
The frequency of usage of clustering algorithms on Alzheimer’s disease data.

Across all of these studies, there are four clustering algorithms used: k-Means, k-Means-Mode, multi-layer clustering, and hierarchical agglomerative clustering (see above sections for description of these clustering algorithms). As Figure 2 shows, hierarchical agglomerative was the most commonly used method throughout the reviewed papers, followed by k-Means and multi-layer clustering and finally k-Means-Mode.

The reviewed studies vary across various dimensions including the clustering algorithm used, the dataset used, variables included in the dataset, and groups included in the datasets (i.e., AD, controls, MCI). Some of the studies have highlighted differences among males and females with AD (Gamberger et al., 2016a,b). Noting that AD is more common in females than in males (Viña and Lloret, 2010; Mazure and Swendsen, 2016), it is possible that there are gender-specific factors underlying the progression of AD in females. The Gamberger et al. studies have highlighted several neural changes between females and males with AD, suggesting that these neural changes may be the underlying reason behind AD being more common in females than in males. Some clustering analyses have shown that AD is not a homogeneous disorder and there are subtypes of AD patients. For example, Noh et al. (2014) have shown that there are three clusters of AD patients that differ in their neural damage. This is important as it may suggest different treatment for each subgroup of patients. Similar findings were also reported in Hwang et al. (2016), thus confirming the existence of subtypes of AD patients. Unlike other clustering studies, Racine et al. (2015) conducted clustering analysis on a dataset that includes individuals at risk for developing AD. The study was able to find several features that explain why some individuals may convert to AD while others do not. These features include low CSF Aβ42 and impaired immediate recall. Cappa et al. (2014) also reported the existence of several subtypes of AD patients that differ in memory and visuospatial impairment. Price et al. (2015) found that there were three groups of AD patients that are characterized by memory, executive dysfunction, or multiple impairments. Similarly, Tosto et al. (2016) found that there are three clusters of AD patients that vary in their extrapyramidal symptoms. According to Armstrong and Wood (1994), AD patients can be subdivided into several groups based on the distribution of senile plaques and neurofibrillary tangles in their brains. McCurry et al. (1999) also reported that there are subtypes of AD patients depending on their sleep disturbances. One problem with the abovementioned studies is that they subtyped AD patients based on very different features varying from neural, cognitive, and clinical variables. Accordingly, it is thus unclear what the subtypes of AD patients are, given the different features reported in every study.

Further, to our knowledge, there were only three studies that have used an MCI population in the clustering analysis (Escudero et al., 2011; Gamberger et al., 2016a,b). Gamberger et al. (2017) found that converting to dementia in individuals with MCI is related to worse baseline cognitive dysfunction as well as having smaller brain volumes. In another study, Gamberger et al. (2016a) found that few individuals with EMCI and some with LMCI were assigned to the same cluster as most AD patients. While the authors did not explain these results, it is possible that these MCI individuals may be at risk of developing AD, and thus were assigned to the AD cluster. Escudero et al. (2011) evaluated several analytic approaches for determining which MCI individuals are likely to convert to AD. They found that by using a large dataset that includes clinical tests and biomarkers in the clustering algorithms, greater accuracy is achieved compared to using smaller numbers of variables in isolation.

Further, to our knowledge, none of the existing studies on clustering analysis have used a dataset that includes early-stage vs. late-stage AD patients. Several experimental studies have shown that these two groups differ profoundly in terms of clinical, cognitive, and neural damage (Kauer-Sant'Anna et al., 2009). Like MCI conversion to AD, clustering analysis can point to several features that underlie the conversion from early-stage AD to advanced AD.

Importantly, while some other medical studies have used semi-clustering algorithms, to our knowledge, there are no studies on using semi-clustering algorithms in AD. While traditional clustering algorithms (as described in this article) work on datasets in which there is no outcome (target) variable nor is anything known about the relationship between the observations (i.e., unlabeled data), semi-clustering enhances clustering by using additional information as constraints in the clustering process. This is helpful in identifying clusters that are linked to a particular target variable. Such additional information is often existent in the dataset or provided by neurologists/clinicians to guide the clustering process. Future work should apply semi-clustering methods on AD.

## Future Research

As mentioned above, only three studies have used an MCI population in the clustering analysis (Escudero et al., 2011; Gamberger et al., 2016a,b). Future research should use more than three populations: healthy controls, individuals with MCI, and AD patients. For example, none of the clustering used subpopulations with MCI, such as amnestic vs. non-amnestic MCI. Such populations are increasingly being studied in the literature, as patients with amnestic MCI are more likely to develop AD than patients with non-amnestic MCI (Mauri et al., 2012; Monacelli et al., 2015).

Another type of clustering is known as fuzzy clustering, in which the classification function causes the class members to become a relative one and an object can belong to several classes at the same time but with different degrees (Ahmadi et al., 2018). Fuzzy clustering has many applications to health sciences, as some individuals may or may not be diagnosed with a certain disorder, depending on different conditions. This is quite relevant to AD. Fuzzy clustering can help us understand the nature of MCI, as some of these individuals may convert to AD, but others may stay healthy.

Further, to our knowledge, different kinds of clustering methods, such as latent profile analysis, were rarely applied to AD datasets. These algorithms do not use a distance function, but instead attempt to produce normally distributed clusters. The latent profile analysis has been applied to several disorders with some success. In one study, Aldridge and Roesch (2008) used latent profile analysis to classify subgroups of adolescents and examine rates of depression and anxiety in these different groups. They observed three clusters of adolescents who vary greatly in their depressive and anxiety symptoms. As another example, Mitchell et al. (2007) used latent profile analysis to subgroup individuals with eating disorders. The analysis revealed five subtypes that have very different profiles. Future research should use latent profile analysis clustering methods to better understand the nature of MCI and their conversion to AD.

## Author Contributions

All authors listed have made a substantial, direct and intellectual contribution to the work, and approved it for publication.

### Conflict of Interest Statement

The authors declare that the research was conducted in the absence of any commercial or financial relationships that could be construed as a potential conflict of interest.
